# Human casein alpha s1 (CSN1S1) skews *in vitro* differentiation of monocytes towards macrophages

**DOI:** 10.1186/1471-2172-14-46

**Published:** 2013-10-02

**Authors:** Stefan Vordenbäumen, Achim Braukmann, Irina Altendorfer, Ellen Bleck, Joachim Jose, Matthias Schneider

**Affiliations:** 1 Heinrich-Heine-University Düsseldorf, Department of Rheumatology, Moorenstr. 5, 40225 Düsseldorf, Germany; 2 Westfälische Wilhelms-University Münster, Institute of Pharmaceutical and Medicinal Chemistry, Correnstr. 48, 48149 Münster, Germany

**Keywords:** Macrophage, Inflammation, Interleukin-1, Interleukin-6, Milk, Differentiation

## Abstract

**Background:**

The milk-derived protein human Casein alpha s1 (CSN1S1) has recently been detected in blood cells and was shown to possess proinflammatory properties. In the present study, we investigated the effect of CSN1S1 on the differentiation of monocytes.

**Methods:**

Primary human monocytes were stimulated with recombinant CSN1S1 and compared to cells stimulated with GM-CSF/IL-4 or M-CSF/IFNγ. Morphological changes were assessed by microscopy and quantification of surface markers of differentiation by FACS analysis. Phagocytic activity of CSN1S1 stimulated cells was measured by quantification of zymosan labeled particle uptake. The role of mitogen activated protein kinases for CSN1S1-induced differentiation of monocytes and proinflammatory cytokine expression was assessed by supplementation of specific inhibitors.

**Results:**

CSN1S1 at a concentration of 10 μg/ml resulted in morphological changes (irregular shape, pseudopodia) and aggregation of cells, comparable to changes observed in M-CSF/IFNγ differentiated macrophages. Surface marker expression was altered after 24 h with an upregulation of CD14 (mean 2.5 fold) and CD64 (1.9 fold) in CSN1S1 stimulated cells. CSN1S1 treated cells showed a characteristic surface marker pattern for macrophages after 120 h of incubation (CD14^high^, CD64^high^, CD83^low^, CD1a^low^) comparable to changes observed in M-CSF/IFNγ treated monocytes. Furthermore, phagocytic activity was increased 1.4 and 1.9 fold following stimulation with 10 μg/ml CSN1S1 after 24 and 48 h, respectively. Early GM-CSF, but not GM-CSF/IL-4 induced differentiation of monocytes towards dendritic cells (DC) was inhibited by addition of CSN1S1. Finally, CSN1S1 induced upregulation of CD14 was impeded by inhibition of ERK1/2, while inhibition of the mitogen activated protein kinases JNK and p38 did not influence cellular differentiation. However, JNK and p38 inhibitors impeded CSN1S1 induced secretion of the proinflammatory cytokines IL-1b or IL-6.

**Conclusions:**

CSN1S1 skews *in vitro* differentiation of monocytes towards a macrophage-like phenotype. Data is accumulating that functions of CSN1S1 are beyond nutritional properties and include immunomodulatory effects.

## Background

Human milk contains numerous proteins with properties beyond nutritional function [1]. Caseins are a main protein constituent of human milk and casein fragments exert a number of biological effects including the modulation of leukocyte adhesion [2], chemotactic properties [3-6], and inhibition of cell growth [7,8] for instance. More recently, a member of the casein family, casein alpha s1 (CSN1S1), was shown to be expressed outside the mammary gland: overexpression was noted in lymph nodes of encephalomyelitic mice and blood of multiple sclerosis patients [9]. Furthermore, independent studies reported overexpression of CSN1S1 in synovial tissue of patients with osteoarthritis and rheumatoid arthritis [10-12]. Consequently, a potential function of CSN1S1 was further characterized by the finding of proinflammatory effects on monocytic cells, like for instance increased expression of IL-1β [13]. Thus, the concept of CSN1S1 as a multifunctional protein with both nutritional and immunomodulatory functions is evolving. Initial events in many inflammatory conditions crucially involve macrophages [14]. Macrophages usually originate from monocytes that are produced in the bone marrow and reach target tissues via systemic circulation [15]. In the present study, we therefore investigated the effect of CSN1S1 on monocytes and possible effects on cellular differentiation *in vitro*.

## Methods

### Blood donors and monocyte isolation

Cells were isolated from 40–60 ml peripheral blood of healthy donors, collected into EDTA tubes (BD Bioscience, Plymouth, UK) by magnetic cell sorting with beaded CD14 antibodies (Miltenyi Biotec, Bergisch Gladbach, Germany). The number of experiments is indicated in the figure legends. Donors had to be free of any medication including over-the-counter drugs, without record of any chronic illness, and currently free of any acute illness such as infections. Cells were seeded out at 1 × 10^6^/ml except for Western Blotting for extracellular signal-regulated kinase (ERK) and c-jun N-terminal kinase (JNK) experiments, cells were seeded out at 3 × 10^6^/ml. The experiments were conducted with the understanding and the consent of each participant. The study was approved by the ethics committee of the medical faculty of Heinrich-Heine-University.

### Cell culture and stimulation experiments

Monocytes were cultured in RPMI 1640 + GlutaMAX™ supplemented with 10% heat inactivated fetal bovine serum, 50 IU/ml penicillin, and 50 μg/ml streptomycin (Invitrogen, Karlsruhe, Germany). 30 μg/ml polymyxin (Sigma-Aldrich, Munich, Germany) was added to experiments to exclude any LPS effects (Sigma-Aldrich) [13], except for the assessment of cellular morphology of living cells, where 200 ng/ml LPS was added in a control experiment. Recombinant human casein alpha S1 (CSN1S1) (Calbiochem, Darmstadt, Germany) was added to cultured cells in indicated concentrations for 24 or 120 h. The following compounds were used to induce *in vitro* differentiation of monocytes as control experiments: M-CSF (R&D Systems, Wiesbaden, Germany) 50 ng/ml, GM-CSF 50 ng/ml, IL-4 20 ng/ml, IFNγ 10 ng/ml (all CellGenix, Freiburg, Germany). For inhibition of casein effects, 20 μmol/l mouse anti human M-CSF antibody (R&D Systems) or cell permeable inhibitors were added as described [13] (all from Calbiochem): briefly, p38 mitogen-activated protein kinase (MAPK)-inhibitor ML3403 was used at 400 nM, ERK 1/2-inhibitor PD98059 was used at 50 μM, JNK-inhibitor (JNK-inhibitor II) was used at 20 μM. Viability of cells was assessed by 3-(4,5-dimethylthiazol-2-yl)-5-(3-carboxymethoxyphenyl)-2-(4-sulfophenyl)-2H-tetrazolium-assay (Promega, Mannheim, Germany) according to the manufacturer’s instructions.

### Phagocytosis assay

Primary human monocytes were seeded out at 1 × 10^6^/ml and stimulated for 24 h with 1 μg/ml CSN1S1 in the presence of 30 μg/ml Px in order to exclude any LPS effects. The uptake of fluorescent labelled zymosan particles was assessed with the colorimetric Cytoselect Phagocytosis Assay (Cell Biolabs, San Diego, CA, USA) according to the manufacturer’s instructions after 24 and 48 h. As a control, cells were cultured in medium including Px only.

### Microscopy

Living cells were photographed at a scale of 400× magnification with Nikon Eclipse TE300 and Nikon Digital Camera DXM 1200 (Nikon, Düsseldorf, Germany) or cells were cultured in chamber slides (Nunc, Rochester, NY, USA), May-Grünwald-Giemsa stained (Merck, Darmstadt, Germany) and photographed at a scale of 200 and 400× magnification with Axioskop 2 Plus (Zeiss, Jena, Germany) and Nikon Digital CameraDS-2Mv (Nikon).

### Flow cytometry

Antibodies were purchased from BD Bioscience (CD14-FITC, CD64-PE, CD83-FITC, CD1a-PE), R&D (CD115-PE), and Biolegend (San Diego, CA, USA: CD116-FITC). After stimulation, cells were incubated with the above antibodies at optimized concentrations. For the assessment of CSN1S1 effects on DC differentiation, primary human monocytes were incubated with 50 ng/ml GM-CSF or 50 ng/ml GM-CSF plus 20 ng/ml IL-4 in the absence or presence of 10 μg/ml CSN1S1. Surface-marker expression was analyzed with FACSort (BD Biosience). Depending on the mean fluorescence intensity, the expression of markers is defined as “low” at < 100 and as “high” at > 100 [15].

### Polymerase-chain-reaction (PCR)

RNA was isolated with Rneasy® Mini Kit (Qiagen, Hilden, Germany), and reverse transcription was performed using QantiTect® Reverse Transcription Kit (Qiagen) according to the manufacturer’s instructions. PCR with real time measurement of fluorescence was carried out on the StepOnePlus Real-time PCR system (Applied Biosystems, Foster City, CA, USA) with 0.3 μM gene-specific, exon-spanning primers for IL-1b [GenBank: NM_000576.2] (Fw: GGGCCTCAAGGAAAAGAATC, Rv: TTCTGCTTGAGAGGTGCTGA) in triplicates using Qantitect® SYBR Green PCR Kit (Qiagen). Results were relatively quantified using glyceraldehyde-3-phosphate dehydrogenase GAPDH [GenBank NM_002046.3] (Fw: CCAGCCGAGCCACATCGCTC, Rv: ATGAGCCCCAGCCTTCTCCAT) as internal and reference RNA (Stratagene, La Jolla, CA, USA) as external standard according to the –ΔΔCT-method.

### Enzyme-linked immunosorbent assay

Quantikine® Human M-CSF-, IL-6- and IL-1-ELISA (R&D Systems) were applied for measuring proteins in the supernatants of cell cultures according to the manufacturer‘s instructions. Determinations were carried out in duplicates. Absorbance was measured at 450 nm using the Anthos 2001 ELISA reader (Anthos Mikrosysteme, Krefeld, Germany).

### Western blot

Western blot was carried out as described before for detection of p38 [16], and JNK or ERK [17]. Briefly, after stimulating primary human monocytes for 24h with 10 μg/ml CSN1S1 total cell proteins were prepared for SDS-PAGE on a 12.5% gel. Electroblotting was carried out onto a polyvinyldifluoride membrane (Porablot; Macherey-Nagel, Düren, Germany). Membranes including the same samples were incubated with either p38-MAP kinase antibody (GeneTex, Eching, Germany) or phosphorylated p38-MAP kinase antibody, JNK or phosphorylated JNK antibody, and ERK or phosphorylated ERK antibody (all Antibodies-online, Aachen, Germany) at optimized concentrations over night at 4°C. After appropriate washing procedures, the membranes were incubated with a 1:10,000 dilution of horseradish peroxidase (HRP)-conjugated anti-rabbit IgG (Sigma). Proteins were visualized via enhanced chemiluminescence (ECL) substrate (Santa Cruz Biotechnology, Santa Cruz, California) and detection by CCD camera (Intas Chemilux ECL Imager).

### Data presentation

Data is presented as error-bars representing mean and standard deviation, representative FACS histograms, or representative photographs of microscopy slides and immunoblots. Data comparison was carried out by two-sided T-test with Bonferroni correction for multiple testing for comparison of surface markers after stimulation with CSN1S1 only, or by one way ANOVA with Bonferroni correction for multiple testing in experiments with inhibitors, antibodies, or when CSN1S1 stimulation was compared to GM-CSF/IL4 or M-CSF/IFNγ stimulation. P < .05 was considered significant.

## Results

### CSN1S1 alters the morphology of monocytes

**Figure 1:**
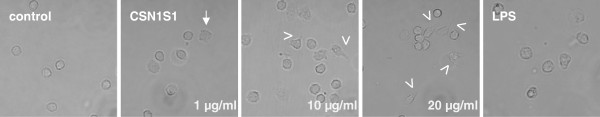
**Live primary human monocytes stimulated with CSN1S1.** Primary human monocytes were incubated with CSN1S1 for 24 h with increasing CSN1S1 concentrations in comparison to medium control and 200 ng/ml LPS. Living cells were photographed at 400× magnification. Cells developed irregular cytoplasm (arrow) and pseudopodia (arrow-head). Representative pictures of 10 experiments.

After incubation of primary human monocytes with recombinant CSN1S1, changes in cellular morphology were noted. Living cells became adherent to the cell culture dish when incubated for 24 h with 1 μg/ml CSN1S1 and the development of pseudopodia was noted in some of the cells when stimulated with higher concentrations (Figure 1). These effects were not observed in the control cultures without CSN1S1, or in case lower concentrations of CSN1S1 (1 ng/ml or 100 ng/ml) were used (data not shown). For comparison, primary human monocytes were incubated with up to 200 ng/ml LPS, which had no visible effect on cellular morphology (Figure 1).

**Figure 2:**
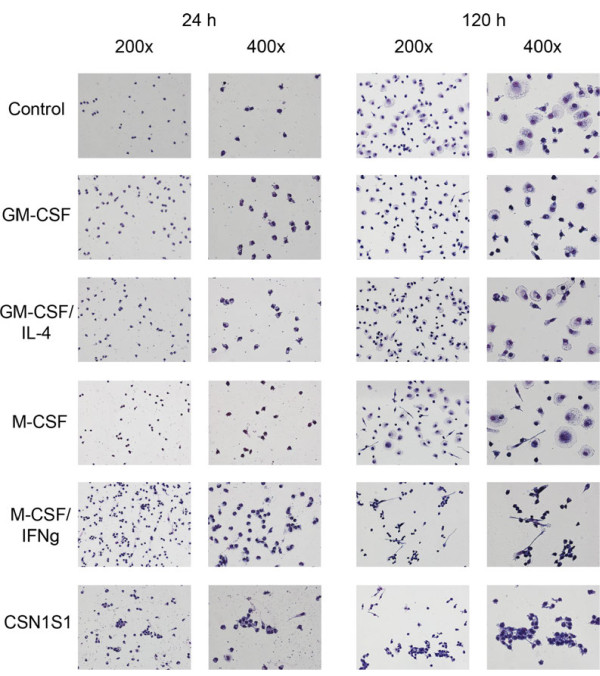
**May-Grünwald-Giemsa stain of primary human monocytes stimulated with CSN1S1.** Primary human monocytes were incubated in medium (control), with indicated cytokines, or CSN1S1 for 24 h or 120 h. CSN1S1 stimulated cells formed aggregates and developed pseudopodia, similar to cells stimulated with M-CSF/IFNγ. Cells were photographed at 200 and 400× magnification. Representative pictures of 10 experiments.

For a better characterization of cellular morphology, primary cells were cultured in chamber slides and stained. Morphology of living cells suggested a differentiation towards DC or macrophages. Thus, we included primary cells stimulated with GM-CSF or GM-CSF/IL4, and M-CSF or M-CSF/IFNγ for comparison to CSN1S1, as the latter stimulants are known to mediate *in vitro* differentiation towards the respective cell types [18,19]. Although we observed rapid morphologic changes in CSN1S1 stimulated cells, *in vitro* differentiation of monocytes is commonly carried out over 5 days. Thus, cells were incubated with the stimulants for both, 24 h and 120 h. As can be seen in Figure 2, after 24 h, control cells were round with little cytoplasm. In contrast, GM-CSF and GM-CSF/IL-4 induced enlarged cytoplasm. M-CSF stimulated cells displayed a smaller increase in cytoplasm with respect to cells stimulated with GM-CSF or GM-CSF/IL4. Moreover, M-CSF, and especially M-CSF/IFNγ treated cells displayed pseudopodia, which were absent in control cells or cells treated with either GM-CSF or GM-CSF/IL4. Furthermore, cells stimulated with M-CSF/IFNγ formed small aggregates. These modifications observed in M-CSF or M-CSF/IFNγ stimulated cells were similar to changes observed in cells stimulated with CSN1S1, which formed aggregates and developed pseudopodia. After 120 h, many control cells became adherent and showed a greatly enlarged cytoplasm. This was also observed for GM-CSF and GM-CSF/IL-4 treated cells. Stimulation with M-CSF caused the development of pseudopodia besides the occurrence of adherent cells with enlarged cytoplasm. The addition of IFNγ to M-CSF again led to a strong tendency towards cellular aggregation and the development of pseudopodia. This was also true for cells incubated with CSN1S1.

### CSN1S1 alters cell-surface marker expression

**Figure 3:**
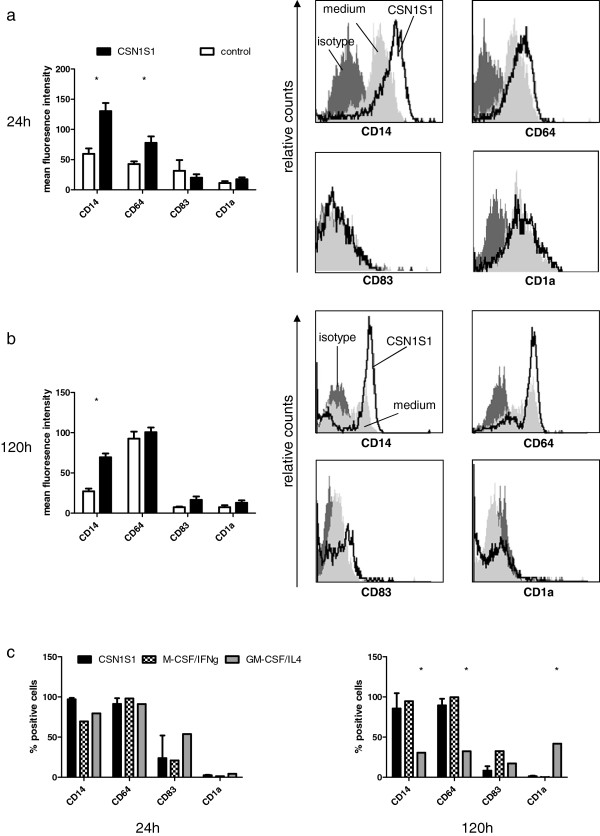
**FACS-Analysis of CSN1S1 stimulated cells.** Surface markers of differentiation in CSN1S1 stimulated monocytes. **a**, **b**. Mean and SD of 6 experiments and representative flow cytometry data after 24 and 120 h of CSN1S1 stimulation or cells kept in medium only (control). * indicates p < 0.05 according to two-sided t-test with Bonferroni correction for multiple testing. **c**. Stimulation with CSN1S1 was compared to differentiation towards macrophages (M-CSF/IFNγ) or dendritic cells (GM-CSF/IL-4) after 24 and 120 h (mean and SD of 6 experiments). * indicates p < 0.05 according to ANOVA with Bonferroni correction for multiple testing.

The cellular morphology of CSN1S1 stimulated cells suggested a differentiation, either into macrophages or into DC. Besides morphological changes, differentiated cells of each type can be distinguished by distinct surface marker expression [18,19]. Therefore, primary human monocytes were incubated for 24 or 120 h with recombinant CSN1S1 and expression of representative surface markers for differentiation of monocytes towards macrophages or DC were analyzed by flow cytometry after immunolabeling. After 24 h stimulation with 10 μg/ml CSN1S1, upregulations of CD14 (2.5-fold ± 1.0, SD) and CD64 (1.9-fold ± 0.8) were detectable (Figure 3a). Lower concentrations of CSN1S1 had no effect (data not shown). The pattern of surface markers obtained was characteristic for macrophages rather than for DC (CD14^high^, CD64^high^, CD83^low,^ CD1a^low^ ) [15,18,19]. After 120 h of incubation with CSN1S1, CD14 but not CD64 remained significantly upregulated. The pattern of surface markers remained the same (CD14^high^, CD64^high^, CD83^low,^ CD1a^low^) (Figure 3b). Next, we compared the surface markers of monocytes differentiated with CSN1S1 to *in vitro* differentiated monocytes towards macrophages (M-CSF/IFNγ) or DC (GM-CSF/IL-4). Such differentiation is known to be obtained by stimulation of primary human monocytes for 120 h [20]. As can be seen in Figure 3c, no difference in surface marker expression was observed after 24 h. In contrast, after 120 h, CSN1S1 and M-CSF/IFNγ stimulation resulted in the same phenotype (CD14^high^, CD64^high^, CD83^low,^ CD1a^low^), while GM-CSF/IL-4 caused a significant downregulation of CD14 and CD64 expression with upregulation of CD1a.

### CSN1S1 increases phagocytic activity of monocytes

**Figure 4:**
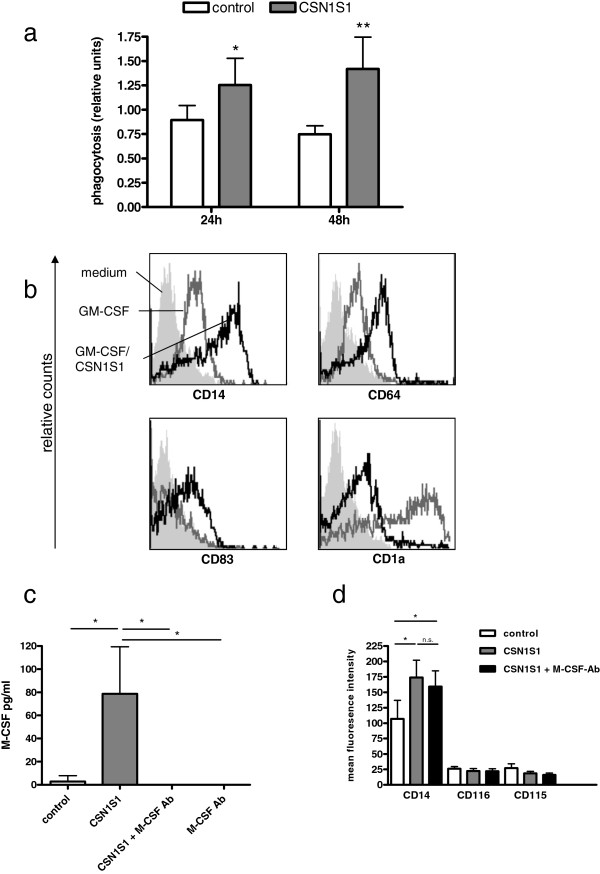
**Characterization of CSN1S1 differentiated monocytes, and the role of M-CSF. a**. phagocytic uptake of labelled zymosan particles in an colorimetric assay of cells stimulated with 10 μg/ml CSN1S1 or medium control (mean and SD of 5 experiments). *, p < .05; **, p < .005, two-sided t-test. **b**. Representative flow cytometry data of monocytes differentiated towards a dendritic cell phenotype by GM-CSF, and reversal of differentiation by addition of CSN1S1 (representative data of 4 experiments). **c**. Stimulation of monocytes for 24 h with 10 μg/ml CSN1S1 in the presence and absence of an anti M-CSF antibody demonstrates neutralization of secreted M-CSF by the antibody (mean and SD of 4 experiments. *, p < .05, t-test). **d**. Effect of M-CSF neutralization by an M-CSF antibody on expression of CD14 and the receptors of M-CSF (CD115) or GM-CSF (CD116) (mean and SD of 4 experiments. *, p < .05, t-test).

Next, we assessed if incubation of primary human monocytes with CSN1S1 also results in functional changes. Increased phagocytic activity is a characteristic property of monocytes differentiated towards a macrophage-like phenotype [18,19]. Therefore, the intracellular uptake of labelled zymosan particles into primary human monocytes was assessed in a colorimetric assay after incubation with 10 μg/ml CSN1S1 for 24 h. There was a marked increase in phagocytic activity of cells after 24 h (1.4-fold ± 0.4), which was sustained after 48 h (1.9-fold ± 0.5, Figure 4a).

### Influence of CSN1S1 on GM-CSF- and GM-CSF/IL-4-induced DC differentiation

The above data suggested that CSN1S1 skews cellular differentiation of monocytes towards a macrophage-like phenotype. We were therefore interested, if an alternative route of differentiation, i.e. early differentiation of monocytes into DC, could be antagonized by the addition of 10 μg/ml CSN1S1 for 24 h. For this purpose, primary human monocytes were incubated with GM-CSF for 24 h in the presence or absence of CSN1S1 and the expression of cell surface markers was assessed by flow cytometry. As can be seen in Figure 4b, GM-CSF alone induced a characteristic immature DC cell surface marker pattern (CD14^low^, CD64^low^, CD83^low^, CD1a^high^) [15]. The addition of CSN1S1 abolished GM-CSF effects and lead to a macrophage pattern (CD14^high^, CD64^high^, CD83^low^, CD1a^low^). Besides GM-CSF, the combination of GM-CSF and IL-4 is a strong stimulus for *in vitro* DC generation [15]. Therefore, we additionally examined the properties of CSN1S1 in influencing GM-CSF/IL-4-induced DC differentiation. GM-CSF/IL-4 similarly caused characteristic immature DC cell surface marker expression (CD14^low^, CD64^low^, CD83^low^, CD1a^high^) after 24 h of incubation, and this effect could not be inhibited by the addition of CSN1S1 (data not shown).

### The role of M-CSF in CSN1S1-mediated cellular differentiation

We previously reported that monocytic cells secrete GM-CSF in response to CSN1S1 [13]. GM-CSF is known to influence the differentiation of monocytes towards a DC phenotype [21]. According to the present results, autocrine stimulation with CSN1S1 induced GM-CSF must therefore be overcome by alternative stimuli to allow for a differentiation towards the observed macrophage-like phenotype. We speculated that autocrine stimulation with M-CSF secreted upon CSN1S1 induction, upregulation of the M-CSF receptor CD115, or downregulation of the GM-CSF receptor CD116 could be responsible for the observed effects. First, primary human monocytes were stimulated with 10 μg/ml CSN1S1 for 24 h and M-CSF secretion into supernatants was quantified by ELISA. As can be seen in Figure 4c, CSN1S1 increased the secretion of M-CSF into culture supernatants 29-fold. As a control, an M-CSF antibody was added to the experiments in order to demonstrate its capacity to bind all secreted M-CSF after stimulation (Figure 4c). In the next step, differentiation of primary human monocytes was induced by 24 h incubation with 10 μg/ml CSN1S1 and the expression of CD14 and the M-CSF and GM-CSF receptors (CD115 and CD116, respectively) were determined by flow cytometry and immunolabeling (Figure 4d). CSN1S1 lead to the expected upregulation of CD14, while the expression of CD115 and CD116 remained unchanged. The addition of an M-CSF antibody to CSN1S1 stimulated primary human monocytes in the same concentration that was demonstrated to bind the secreted M-CSF protein (Figure 4c) did not alter the expression of CD14 or the receptors CD115 and CD116. Thus, neither changes in the expression of M-CSF, nor up- or downregulation of M-CSF receptor (CD115) or GM-CSF receptor (CD116) respectively, explained the preferential shift of monocyte differentiation towards macrophages in culture conditions that contain both M- and GM-CSF.

### CSN1S1 induced differentiation and cytokine expression may partially be mediated via MAPK

**Figure 5:**
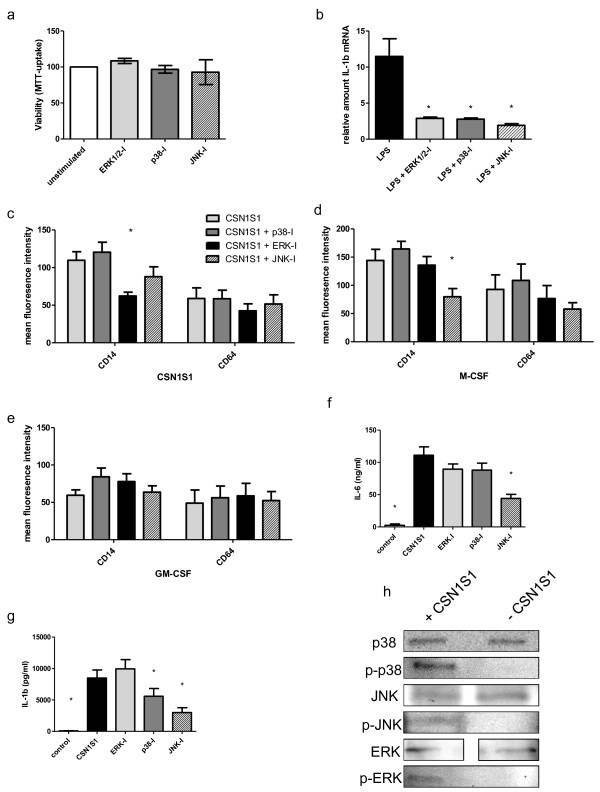
**The role of MAPK in CSN1S1 induced cellular differentiation. a**. Effect of ERK1/2-, p38-, and JNK-Inhibitor (I) on cellular viability of primary human monocytes. **b**. Significant suppression of LPS-induced IL-1b-mRNA production by addition of ERK1/2-, p38-, and JNK-I (mean and SD of 4 experiments, p < 0.05, t-test). **c**-**e**. surface marker expression in primary human monocytes stimulated with 10 μg/ml CSN1S1 **(c)**, M-CSF **(d)**, and GM-CSF **(e)** for 24 h in the presence of indicated MAPK-I (mean and SD of 4 experiments. * p < 0.05, ANOVA with Bonferroni correction). **f**, **g**. Secretion of IL-1b **(f)** and IL-6 **(g)** protein into supernatants of primary human monocytes stimulated with CSN1S1 and indicated MAPK-I (mean and SD of 4 experiments. * p < 0.05, ANOVA with Bonferroni correction). **h**. Western-blot of unphosphorylated and phosphorylated (p-) p38-, JNK, and ERK-MAPK molecules in cell lysates of primary human monocytes stimulated for 24 h with 10 μg/ml CSN1S1. Images from P38 blots are separated, because protein samples were loaded in two pockets separated by an empty pocket due to blending of bands when adjacent pockets were used.

We previously reported that CSN1S1 upregulates the expression and secretion of GM-CSF in monocytes in a p38 MAPK-dependent fashion [13]. We were therefore interested to analyze if cellular differentiation and the expression of other proinflammatory cytokines is also dependent upon MAPK pathways. In a first step, we analysed if addition of inhibitors of the MAPK pathways, i.e. JNK, p38, and ERK, influenced overall survival of primary human monocytes. As can be seen in Figure 5a, there was no significant effect on cellular vitality of monocytes by addition of inhibitors for 24 h in the concentrations used in subsequent experiments. Next, we assessed if the addition of these inhibitors was biologically effective in suppressing MAPK mediated signalling. LPS-signalling is known to be mediated via all three MAPK: JNK, p38, and ERK, and results in IL-1b expression [22]. Therefore, primary human monocytes were stimulated with LPS for 24 h in the presence and absence of MAPK inhibitors and IL-1b-mRNA expression was measured as a control experiment. As can be seen in Figure 5b, all inhibitors significantly suppressed IL-1b-mRNA expression to a similar degree. In order to identify a putative signal transduction mechanism responsible for CSN1S1-induced cellular differentiation, we then tested the ability of MAPK inhibitors to impede the generation of a macrophage-like phenotype. Primary human monocytes were therefore incubated with MAPK inhibitors before stimulation with 10 μg/ml CSN1S1. Cell surface markers CD14 and CD64, upregulated during CSN1S1-induced differentiation as described above, were assessed by flow cytometry and immunolabeling. As depicted in Figure 5c, CSN1S1-mediated upregulation of CD14 was significantly decreased by inhibition of ERK, but not p38 and JNK. Next, we were interested to see if this effect was specific for CSN1S1 stimulated cells or if ERK-inhibition generally reduces CD14 expression in monocytes treated with M-CSF or GM-CSF. To this end, cells were treated with MAPK inhibitors prior to stimulation with M-CSF. As shown in Figure 5d, upregulations of CD14 in M-CSF treated cells were not influenced by inhibition of ERK as in CSN1S1 treated cells, but by inhibition of JNK. In contrast, MAPK inhibition did not influence surface marker expression in cells stimulated with GM-CSF for 24 h (Figure 5e). This suggests that signal transduction of CSN1S1-mediated differentiation is at least in part distinct from pathways used by M-CSF.

In order to evaluate whether CSN1S1 induces the expression of proinflammatory cytokines via the same route, primary human monocytes were stimulated with CSN1S1 and IL-1b and IL-6 protein-secretion into culture supernatants was measured in the presence and absence of MAPK inhibitors. As can be seen in Figure 5f-g, a significant reduction in the upregulation of IL-1b was noted with inhibition of p38 and JNK while IL-6 was decreased with inhibition of JNK only. Of note, inhibition of ERK did not reduce increased cytokine secretion in a significant manner.

The above results suggested that CSN1S1 engages all 3 MAPK molecules to either exert effects on cellular differentiation or proinflammatory cytokine expression. To confirm this notion, we additionally assessed activation, i.e. phosphorylation, of MAPK after incubation of primary human monocytes with 10 μg/ml CSN1S1 for 24 h by Western blot. As can be seen in Figure 5h, p38, JNK, and ERK were all phosphorylated when stimulated with CSN1S1, but not in control experiments without CSN1S1.

## Discussion

In the present study, we demonstrate that exposure of primary human monocytes to CSN1S1 *in vitro* consistently skews cellular differentiation towards macrophages, including morphological changes, distinct surface marker expression, and functional properties such as increased phagocytic activity. Additionally, CSN1S1 induces the expression of proinflammatory cytokines [13]. Besides these functions, the most obvious role of human CSN1S1 is to provide an amino acid source to the new-born. However, the acquisition of additional functionality in an evolutionary context is an increasingly recognized phenomenon, also referred to as protein promiscuity [23,24]. In accordance with this notion, caseins are considered to have arisen from innate immune genes, and that their nutritive functions are a consequence of a more recent evolutionary development [25]. This assumption is based on the conserved organization of the casein genes in a cluster of innate immune genes that also includes the histatin/statherin-family [25]. The hypothesis that CSN1S1 is a multifunctional protein is further supported by its state as a disordered protein with multiple potential tertiary conformations [26]. This last point has to be regarded with caution however, since – to the best of our knowledge – crystallographic analyses of the human CSN1S1 structures currently do not exist. Since CSN1S1 is not only an endogenous produced protein, but is also a component of milk, the question arises, which potential functions CSN1S1-induced IL-1β expression could have in the offspring. Intestinal exposure to antigens and milk constitutes an important trigger for the development of a competent immune system in the new-born [27]. It is therefore interesting to speculate that CSN1S1 in mother milk may contribute to the development of a patent immune system by triggering immune responses to potential pathogens by activation of innate immune responses like for instance IL-1β secretion. Moreover, CSN1S1 by itself gives rise to sustained specific IgG antibody production in nursed individuals [28]. Early infantile autoantibody production in turn is speculated to confer protection to pathogens [29]. On the other hand, there are several mechanisms which could potentially prevent overwhelming inflammation triggered by exposure to CSN1S1 in milk: CSN1S1 is only a minor component of human milk and constitutes approximately 5% of the casein-fraction [30]. Moreover, CSN1S1 may be degraded by proteases in the healthy gut, thereby preventing IL-1β induction. Further research is clearly warranted to clarify these exciting new hypotheses and to explore, if variations in CSN1S1 exposure or extra-mammary expression may contribute to defective immune reactions. The recent findings of CSN1S1 overexpression in the autoimmune diseases multiple sclerosis and rheumatoid arthritis [9,10,12] may be considered supportive of this hypothesis.

In the present experiments, the effect on all aspects of cellular differentiation, i.e. change of morphology, surface marker expression and increased phagocytosis, were observed rapidly, within 24 h of stimulation. Furthermore, CSN1S1 was able to reverse early GM-CSF-induced monocyte differentiation into DC, resulting in a macrophage like phenotype. *In vitro* differentiation of monocytes towards macrophages or DC is most commonly carried out over 5 days, although more rapid differentiation in the course of several hours is recognized depending on the stimulus used [20]. In accordance with this notion, characteristic differences between *in vitro* differentiation towards macrophages (using M-CSF/IFNγ) or DC (using GM-CSF/IL-4) were observed after 120, but not 24 h. Of note, surface markers were strikingly similar between M-CSF/IFNγ and CSN1S1 treated cells. However, CSN1S1 failed to reverse *in vitro* generation of early DC by a combination of GM-CSF and IL-4. This may be due to the more potent effect on *in vitro* DC generation by the combined cytokines compared to GM-CSF alone [15,21,31].

We were consequently interested to explore potential mechanisms employed by CSN1S1 to induce monocyte differentiation and cytokine expression. It was previously reported that primary human monocytes secrete GM-CSF in response to CSN1S1 [13]. This was somewhat puzzling, because GM-CSF is known to influence the differentiation of monocytes towards a DC phenotype [21]. On the other hand, according to the present data, CSN1S1 does also increase the secretion of M-CSF into culture supernatants. However, addition of a neutralizing M-CSF antibody to stimulated monocytes did not abrogate CSN1S1-effects. Importantly, there were also no changes in expression of the GM-CSF- or M-CSF-receptors (CD115 or CD116, respectively). Thus, CSN1S1 likely induces its effects on monocyte differentiation by a mechanism independent from M-CSF signalling. Concerning intracellular messengers, CSN1S1, like other proinflammatory cytokines such as IL-32 for example, employs p38 MAPK to induce proinflammatory cytokine expression [13,19]. Inhibition of another member of the MAPK family, ERK1/2, a well-known regulator of cellular differentiation [32], but not p38 or JNK led to a decrease in CSN1S1 induced upregulation of CD14 in the present experiments. This effect may be specific for CSN1S1 rather than attributable to the process of differentiation of monocytes towards macrophages in general, because M-CSF induced upregulation of CD14 was inhibited by JNK exclusively. Furthermore, in contrast to differentiation, the secretion of proinflammatory cytokines (i.e. IL-6 and IL-1b) was influenced by the inhibition of JNK and/or p38, but not by ERK1/2. It cannot be excluded that other second messengers are employed for CSN1S1 induced cellular differentiation as well, especially because CD64 was not significantly affected by ERK1/2 inhibition. In conclusion, the data suggest that MAPK may be differentially involved in mediating CSN1S1 induced effects on cellular differentiation or cytokine expression. Further research in this direction is warranted however, before firm conclusions can be drawn.

A limitation to the study consists in the fact that the concentration of CSN1S1 in potentially relevant tissues for monocyte differentiation such as e.g. inflamed nerves, joints, or even the gastrointestinal tract is unknown. In order to simulate physiologic conditions, the concentrations of CSN1S1 used in the present experiments was determined based on previous observations: While *in vitro*-experiments suggest that ectopic CSN1S1 secretion by monocytes is in the range of ng/ml [9,13], human milk contains 2.4 mg/ml total casein [33], approximately 5% of which is made up of CSN1S1 [30]. This results in a concentration of 120 μg/ml CSN1S1. Although proteins contained in milk are exposed to proteases within the digestive tract, they may be absorbed in an intact form which is even favoured by immature digestive functions of infants and protease inhibitors within milk [34]. Thus, concentrations used in the present experiments may reflect local conditions *in vivo*.

## Conclusions

Human CSN1S1 influences the differentiation of monocytes towards macrophages *in vitro* and mediates the expression of proinflammatory cytokines. This process is at least partially dependent on differential MAPK signalling. The notion of CSN1S1 as a multifunctional protein with immunomodulatory properties beyond nutritional aspects is further evolving.

## Abbreviations

CSN1S: Human casein alpha S1; GM-CSF: Granulocyte-macrophage colony-stimulating factor; M-CSF: Macrophage colony-stimulating factor; FACS: Fluorescence activated cell sorting; MAPK: Mitogen associated protein kinase; ERK1/2: Extracellular signal-regulated kinase 1/2; JNK: c-Jun N-terminal kinase; DC: Dendritic cells.

## Competing interests

SV, AB, IA, EB, JJ, MS report no conflict of interests.

## Authors’ contributions

Conceived the study: SV, JJ, MS. Data acquisition: SV, AB, IA, EB. Data analysis and manuscript preparation: SV, JJ, MS. All authors read and approved the final manuscript.
